# Visual search for people wearing protective clothing in a forestial environment – Differences between gaze and behavior

**DOI:** 10.1371/journal.pone.0344577

**Published:** 2026-03-10

**Authors:** Florian S. Oswald, Wolfgang Einhäuser

**Affiliations:** Physics of Cognition Group, Chemnitz University of Technology, Chemnitz, Germany; LV Prasad Eye Institute, INDIA

## Abstract

To prevent hunting accidents, people in forests should be well-visible to others. However, there is debate which kind of protective clothing should be required. We created a database containing photographs of 22 individuals in forestial settings. The database is available online; for the present study a subset of images was used. The photographed persons (“models”) wore four different clothing variants adding to typical forest garment: Two conditions included protective clothing, either a reflective vest or a hat with reflective hatband. We included two conditions without protective clothing, either with a hat (without hatband) or with no hat, and a condition without person. For the present study, we cropped and scaled the image of the database to generate 800 stimuli (160 per condition). These were presented to N = 16 observers, who were asked to respond as quickly as possible without sacrificing accuracy whether a human was present. Reaction times (RTs) and miss rates depended on clothing. The two conditions without protective clothing had indistinguishable error rates, while fewer errors occurred with hatband, and even fewer with the vest. RTs were faster with protective clothing than without. In the absence of protective clothing, responses were faster without hat. Interestingly, vest and hatband led to indistinguishable RTs. However, individuals in vests were looked at earlier than individuals without. This difference between RTs and fixation latencies is likely explained by a frequent additional saccade from vest to face before the response, presumably to verify the human presence. In conclusion, although RTs suggest no advantage for wearing reflective vests over hatbands, error rates and eye-tracking data reveal a potentially critical safety benefit of wearing reflective vests in forestial environments. More generally, our study demonstrates the benefit of combining eye-tracking data with behavioral measures using a real-world example.

## Introduction

In 2006, Dick Cheney, then US vice president, famously shot at and wounded a man during quail hunting, apparently for failing to notice him approach from the side while taking aim at birds [1]. There is plenty of anecdotal media coverage on similar incidents – often with tragic outcomes. As only a small fraction of firearm-related accidents relates to hunting (e.g., 1.95% in the United States from 1993 to 2008 [2]) and conversely only a small fraction of hunting accidents relates to firearms [3], quantitative data on the causes of firearm-related hunting accidents is scarce. A survey on firearm-related hunting accidents in Germany between 1961 and 1992 [4] reports that out of 257 analyzed cases, “failure to notice the victim” and having “mistaken the victim for game” account for about a third of such accidents (24.1% and 9.3%, respectively). Hence, good visibility seems a critical protective factor. Indeed, recommendations for operating in hunting areas typically include wearing protective clothing. For example, the US Fish and Wildlife Service recommends to “Wear bright clothing (like blaze orange).” to “make yourself easily visible.” (https://www.fws.gov/story/tips-hunters-and-non-hunters). In practice, however, recreational hunters frequently refrain from wearing full body reflective garment, and rather resort to alternative means. A particularly common alternative are reflective hatbands, even though some regional hunting regulations explicitly rule them to be insufficient for specific hunting activities (e.g., Article 52 in [5]). This raises the questions whether such hatbands increase visibility in forestial settings, and, if so, how they compare to reflective vests.

While little research has addressed reflective vests and similar protective gear in forest environments, there is a substantial body of literature on reflective clothing for workers and for vulnerable road users (VRUs; e.g., pedestrians and cyclists). The notion that wearing clothing that is well visible for drivers would increase VRUs’ safety in traffic and its scientific study date back to at least the mid-20^th^ century [6]. With the increasing availability of wearable reflective materials, numerous studies on VRUs have compared different versions of reflective clothing. For example, in a simulated night driving experiment (the participants sat in the passenger seat and reacted to cardboard pedestrians), a substantial visibility advantage was found for reflective vests over no vests with substantial differences between reflective stripe colors [7]. While it seems evident that visibility is helped by reflective clothing, how exactly increased visibility translates into actual safety benefits is harder to quantify [8]. Recently, there has been a shift from merely detecting a pedestrian (i.e., making them visible) to recognizing them as pedestrians (i.e., making their motion visible) [9]. In this context, placing reflective markers at the extremities (and thereby making the biological motion apparent) is more effective than placing the same markers at the torso [10]. Such improvements of recognizability as pedestrians are particularly critical for the visibility to older drivers and to drivers with limited motion perception capabilities [11]. Despite the obvious benefits in visibility and the likely resulting benefit to safety, the question whether to wear protective clothing – not only for pedestrians but also for workers – is often perceived as trade-off between safety and perceived comfort, which presents a challenge to regulators and clothing manufacturers [12,13]. In any case, assessing (static) visibility of different clothing options is arguably the first step towards safety recommendations, which is our rationale to investigate visibility in the forest with static scenes and a visual-search task.

To investigate visibility, we created a database of people standing in forestial settings while wearing different pieces of clothing, in particular reflective vests and hatbands. We used this database to generate stimuli for a visual-search task, in which observers had to decide as quickly as possible whether a human was present in the scene. Visual search is one of the most widely used paradigms in experimental psychology, which dates back to the seminal work by Treisman and Gelade in the early 1980s [14]. While their distinction of parallel search (for individual features) from serial search (for conjunctions of features), might be overly simplified with respect to the underlying mechanisms [15], the task to make a rapid decision as to whether a certain item is present in a scene has transcended simple search arrays and is nowadays widely used in the context of natural objects, scenes and in applied research [16]. For the present purpose, we consider visual-search performance (accuracy and reaction time) a good proxy to assess visibility.

While most studies on visual search in real-world scenes are concerned with how scene structure (or scene grammar, [17]) guide attention, our present approach is motivated by the applied question. The stimuli therefore contain mostly foliage and provide limited contextual guidance beyond the fact that humans stand on the ground, which is readily visible in some scenes, though not in all. This provides some guidance in the vertical direction [18], but very little in the horizontal. We further limit spatial guidance by cropping different regions of the same scene, such that the target appears at a random location (with some constraints) relative to the screen. While the resulting repetition of the same basic scene (i.e., the same target in the same context, but at different screen locations) may provide some additional guidance, the randomization of spatial location along with presenting scenes and their mirrored versions likely outweighs any such effect. Hence, we consider our study an example of naturalistic visual search with low contextual guidance.

In addition to contextual guidance, several visual factors impact visual-search performance. During free viewing, scene regions that differ from their surround in terms of features like luminance, color or orientation (i.e., are visually “salient”), are preferentially attended [19], although this does not imply that these low-level features are *causally* involved in guiding attention [20]. When searching for an item unrelated to the scene, the effect of salience on attention is diminished or absent [21,22]. However, this does not mean that the salience of the *search target* would be irrelevant; for example, when potential target locations are contextually constrained (e.g., search for a pedestrian, who will likely occur near the ground), search performance is well predicted by a combination of salience and such prior knowledge [18]. In natural scenes, salience correlates with the interestingness of a location [23,24] and with its semantic meaning. Indeed, semantic meaning – modelled based on the judgement of independent observers – explains away the effects of salience during free viewing [25]. The thus defined meaning is also predictive of visual search performance for objects in natural scenes [26]. The probability to find an object (or to look at it in free viewing) also depends on spatial factors, such as its distance from the scene center [27], which in some case can explain away effects of salience [28], though not of meaning [29]. Object size is another factor that influences attention: in search arrays, a task-irrelevant larger item can act as additional singleton and capture attention [30]; for free-viewing, larger objects are looked at more likely than smaller objects in natural scenes [31]. Importantly, attentional guidance does not only depend on retinal size, but also on apparent size [32,33] and – in natural scenes – the size expected based on perceived distance [34]. For the present experimental context, these observations necessitate that in the design of the stimuli size and distance (both in relation to the image and with respect to the actual distance and size in the depicted size) should be equated as well as possible across the different conditions of interest.

Although we provide observers with no instruction regarding gaze other than to fixate centrally before each trial, we recorded eye movements throughout, as gaze data may provide insight into search strategies that are unavailable with response time data alone [35]. Moreover, effects measured based on the timing of gaze can be distinct from reaction times measured behaviorally, as the latter not only include search *per se* (“scanning”), but also target verification (for the distinction of search initiation, scanning and verification, see ref. [36]).

The aim of the present study is two-fold. First, it addresses the applied question whether reflective vests provide an advantage over hatbands for being seen in a forest, and whether either piece of clothing provides advantages over no protective clothing. While we hypothesized a benefit of the protective vests over no protective clothing, we considered the question of the efficacy of the hatband open. Second, the study provides general insight into naturalistic visual search in the absence of strong spatial guidance, including a potential dissociation between behavioral responses and gaze patterns.

## Methods

### Observers

Sixteen volunteers (4 men, 12 women, age: 19–35, mean: 23.2 years, sd: 4.1 years) participated. All had normal or corrected-to-normal visual acuity and normal color vision as assessed by a Snellen chart and Ishihara plates, respectively. All gave written informed consent prior to participation and received either course credit or 10 EUR/hour as compensation. Sample size was determined under the assumption of a large effect (d_Z_ = 0.8) for the pairwise comparison of interest – i.e., whether or not the mean of the median reaction times differs between two clothing conditions – for a power of 80% at an alpha level of 0.05. This yields a sample size of at least 15. N = 16 was chosen as an even number was required for counterbalancing the response mapping. The assumed large effect size and the resulting sample size is in line with many similar studies of visual search (e.g., sample sizes between 10 and 15 in each experiment of [37], of 15 and 16 in each experiment of [38], and between 5 and 20 in [15]).

### Apparatus

Stimuli were presented on a ViewPixx/3D Full LCD display (VPixx Ltd., Saint-Bruno, QC Canada) located 57 cm from the observers and running at 1920 × 1080 pixels@120 Hz. Observers responded by pressing the left and right button of a ResponsePixx (VPixx Ltd.) button box with their left and right index finger, respectively. Observers’ gaze direction was measured monocularly at 1000 Hz using an Eyelink-1000 (SR Research Ltd., Ottawa, Canada) eye tracker. Observers’ head was stabilized with the padded forehead rest and chin rest that is part of the Eyelink equipment. Stimulus presentation and eye tracking was implemented in Matlab with its Psychophysics and Eyelink toolboxes [39–41]. The experiment was conducted in a sound-attenuated room specifically designed for psychophysical experiments, in which the stimulus monitor was the only light source.

### Database

**Fig 1:**
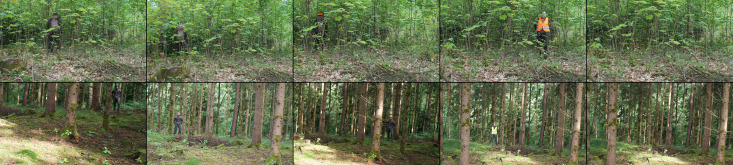
Example stimuli. *From left to right:* hat, no hat, hatband, vest, target-absent; note the matched target size within each set, while there is variability between locations; for both examples, the target-absent scene matching the vest scene is depicted (these are distinct photographs, no image manipulation besides cropping was used in any case throughout this study). *All persons depicted agreed in writing to the unrestricted use of their image, in particular to inclusion in the online database and to the use in psychophysical experiments. All images have been made available by the authors under a CC-BY-4.0 license.*

Prior to the experiment, we created an image database of 22 volunteers (hereafter: “models”), each photographed standing at a different forest location in four different clothing conditions (without hat, with hat, with reflective hatband, with reflective vest) at 4912 × 2760 pixels resolution. Otherwise, models wore dark clothing, typical for the forestial environment (Fig 1). One model was an author, the images of the other 21 models were used for the experiment – 20 for the main part, 1 for training. No model was included as observer. As the photographs were taken about 350 km from the experiment site, most models were unknown to the observers, though three observers reported after the experiment to know one of the models in person. In addition, an image without person was photographed at each of the locations, which is used to create the “target-absent” stimuli for the present study.

Moreover, four additional photographs were included in the database per site (i.e., per model) that were not used for the present study, but might be of interest for future research: three of these contain no person, but one piece of garment (i.e., the hat with hatband, the hat without hatband, the vest) in approximately the same location as the person was located and one contains the person with vest and hatband holding a color reference chart. The full database is available at https://doi.org/10.17605/OSF.IO/CGS6X and contains additional data for each image (distance between camera and object/person, exposure duration, aperture, the ISO-equivalent sensitivity, date and time of recording, camera viewing direction, wind speed and direction). All models agreed in writing to the use of their pictures in psychophysical experiments and to making them publicly available without constraints. The chosen locations were on publicly accessible grounds without any restrictions to photography.

### Stimuli

**Fig 2:**
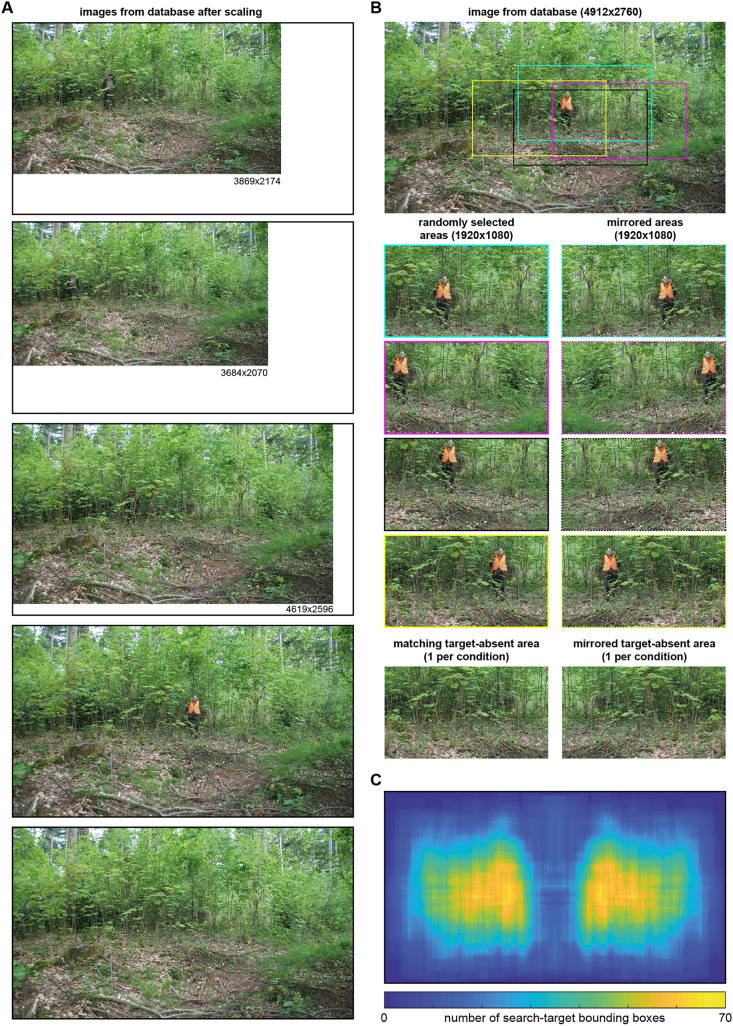
Creation of stimuli from database images. **A**) Images from the database (original resolution 4912 × 2760 pixels) for the same model (#2 in the database); target-present images are scaled down such that the target size is identical across conditions using the smallest bounding box for the model as reference; the reference image (here the vest condition, second panel from bottom) remains unscaled; black frames are to ease size comparison and not part of the images. **B**) The eight target-present stimuli created from the example image on top (also forth image of panel A). The stimuli on the right are the mirror reflected versions of the stimuli on the left. The target-absent stimuli are created by randomly picking one of the cropping areas and applying it to the target-absent image taken at the same location as the model’s images (bottom of panel A), resulting in a total of 8 target-absent images per location; hence, there are a total of eight images of each condition per location (model). **C**) Histogram of the locations of the models’ bounding boxes (i.e., search targets) relative to the stimulus (and screen) for all 640 (20 models×4 conditions×8 versions) target-present stimuli. *The person depicted in panels A and B agreed in writing to the unrestricted use of their image. All images have been made available by the authors under a CC-BY-4.0 license.*

Using models 01 through 20 from the database, we created 800 unique stimuli (Fig 1): For each condition, we manually marked the bounding box of each model in each target-present condition. The four images of each model were then scaled such that the bounding box width corresponded to the minimum of the four bounding boxes (Fig 2A). That is, for each model, the image for one condition was not scaled, while the others were downscaled for the persons to match in width. For one of the models (person 18 in the database), 40% rather than the minimum was used (requiring one condition to upscale), as otherwise the subsequent requirements could not be met. For all other models, scaling ranged between 47.1% and 100% (mean: 81.5%). From each of the 80 images (4 conditions × 20 models), four distinct regions of 1920 × 1080 pixels were cropped (Fig 2B), which served as stimuli for the present study. The purpose of the cropping was to accommodate the stimulus to the screen resolution, to insert variability of the search target’s location relative to the screen, and to keep the size of the search target identical for all conditions of one model. The cropping area was selected at random, provided that (i) it was fully within the image and (ii) the center of the person’s bounding box had a distance of at least 180 pixels (5 degrees of visual angle [dva]) from the image center. The latter constraint was relaxed to 72 pixels (2 dva) for the hat condition of person 18. Each of these images was used as is and reversed at the vertical midline. This yielded 160 stimuli per target-present condition (20 models × 4 instances × 2 mirror reversals). For each model and target-present condition, we created a target-absent stimulus by applying the same scaling and the same shift as for one of the target-present instances to the corresponding target-absent photo. This target-absent stimulus was then also mirror reversed such that we have a total of 160 target-absent images (20 models [in whose setting the target-absent image was taken] × 4 conditions [to which the scaling is matched] × 2 mirror reversals). In the vest condition, half of the models wore orange vests, half wore yellow vests. The same procedure was applied to images of the model 21, the resulting stimuli were used for an instruction block prior to the main experiment. Besides the scaling, cropping and mirroring no other image manipulation was applied; in particular, never was a search target (model) moved artificially to a different position than the one it was standing at in the real world.

Despite the matching of width through scaling and the distance constraints, the exact area and location of the target model in the forest scene varied between conditions. Since larger size and smaller distances to the image center might ease search, we tested whether there were systematic differences in these variables between the target-present conditions. To this end, we averaged the area of the bounding boxes for the eight different stimulus versions of each model as well as the Euclidian distances between the centers of the bounding box and image. (As the mirrored images are identical in these respects, each average effectively includes four numbers). The resulting numbers were then subjected to a repeated-measures analysis of variance (rmANOVA) with the 4-level factor condition and treating the models as subjects for this purpose. We found no evidence for a dependence of area (F(3,57)=0.36, p = .781, ɳ^2^ = .019) nor of distance (F(3,57)=0.75, p = .528, ɳ^2^ = .038) on condition.

### Procedure

Each observer was presented each of the 800 stimuli once, the order of presentation was random with a different order for each observer. Each trial started with a black central fixation cross on a gray background, whose luminance of 49 cd/m^2^ corresponded to half the maximum luminance (“white”) of the images. Once observers had fixated within 1 dva of the cross for 300 ms, the stimulus was presented. The observer’s task was to indicate by a button press as quickly as possible without sacrificing accuracy whether a person was present in the scene. They were explicitly instructed that they were allowed to move their eyes naturally when the scene is present. The assignment of buttons (left/right) to the response (target-present/target-absent) was counterbalanced across observers. Before the first trial, the eye-tracker was calibrated with a 13-point calibration grid and the calibration was validated with a similar grid. Whenever observes failed to fixate the pre-trial fixation cross for 5s or when 50 trials since the last calibration/validation had elapsed, the eye tracker was recalibrated. Prior to each calibration, observers were encouraged to take a break. Before the main experiment, observers completed a short familiarization block that consisted of eye-tracker calibration/validation and five trials (one per condition, the four target-present trials with images of model 21, which was not used in the main experiment).

This research complied with the tenets of the Declaration of Helsinki and was approved by the Institutional Review Board at Chemnitz University of Technology (*Ethikkommission der TU Chemnitz*; case no. #101699210). Informed consent was obtained from each participant. All agreed to making their data publicly available in anonymized form. All experimental data are available at https://doi.org/10.17605/OSF.IO/Z723N. Data collection took place at TU Chemnitz from July 5^th^ to July 30^th^ 2024.

### Analysis

Since it could be expected a priori that target-absent trials take substantially longer to complete than target-present trials and our interest is in the differences between the target-present conditions, most statistical analyses pertained to target-present trials alone. We considered three main dependent variables (DVs), the error rate (percentage of incorrect trials per participant and condition), the behavioral reaction time for correct trials (the median time between image onset and button press per participant and condition) and the time to the first fixation of the target’s bounding box for correct trials (the median time for trails in which the target was fixated per participant and condition). The effect of condition (four levels: hat, no hat, hatband, vest) on each DV was assessed by a 1-factor rmANOVA.

For assessing observers’ response criteria and sensitivity, we computed signal-detection-theory (SDT) measures c and d’, aggregating target-present trials across all conditions. Since hit rates were near ceiling and false alarm rates near floor, we applied the correction suggested in [42]. This is, corrected hit rates were computed as:


hitRate =(#hits + 0.5) / (#hits + #misses + 1)

where #*hits* is the count of target-present trials to which a participant responded “present” and #*misses* is the count of target-present trials to which the participant responded “absent”. Corrected false-alarm rates were computed as


faRate=(#falseAlarms+0.5)/(#falseAlarms+#corRej+1)

where #*corRej* (correct rejections) is the count of target-absent trials to which the participant responded “present” and #falseAlarms is the count of target-absent trials to which the participant responded “present. Using these corrected values, the sensitivity d’ and the criterion c were computed as usual:

d’ = z(hitRate) – z(faRate)

c = – 0.5 (z(hitRate) + z(faRate))

d’ and c were computed per individual participant.

Since the task could be performed without looking at the target, we decided prior to analysis that only participants that looked at the target in each condition for at least 50% of the trials would be included in the gaze-based analysis. As detailed in the section “Results – Gaze”, this led to the exclusion of the data of one observer.

## Results

### Behavior

#### Accuracy.

**Fig 3:**
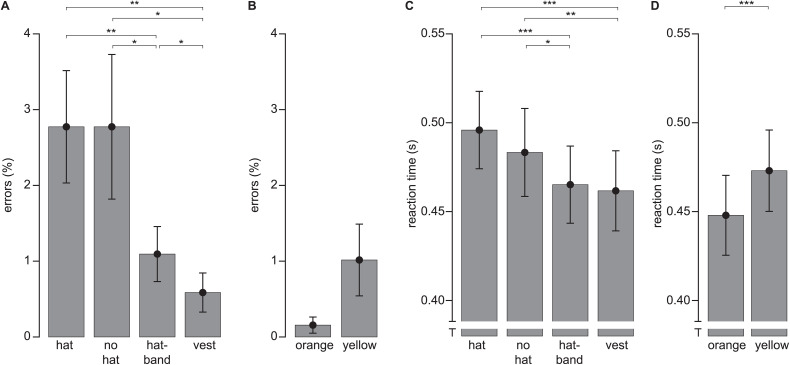
Behavioral Data. **A**) Error rate by condition (only target-present conditions shown, target absent at 1.8%); **B**) Error rate for vest condition, split by vest color; **C**) Behavioral reaction times (RTs) by condition, target-absent data at 1.01s not depicted; **D**) RTs for vest condition, split by vest color. All bars denote mean and standard error of the mean (s.e.m.) across individuals. Significance markers in all panels refer to pair-wise comparisons (*p < 0.05; **p < 0.01; ***p < 0.001).

Overall, participants made only few errors in their present/absent judgements. In target-absent trials, error rate was 1.8% (standard deviation [SD] across observers: 1.1%); that is, in 1.8% of trials observers incorrectly reported a person to be present in the image. For target-present trials, the error rate (no person reported despite presence of a person) depended on the condition (Fig 3A; F(3,45)=8.65, p < .001, ɳ^2^ = .366). Error rate was highest for the conditions without protective clothing (hat and no-hat), which were indistinguishable (t(15)=0, p = 1, d_Z_ = 0 [Incidentally, the means are exactly identical: aggregated over all participants there were 71 error trials of 2560 (160 stimuli × 16 participants) trials per condition, yielding a 2.77% (71/2560) error rate in both conditions)]. Error rates were lower with the hatband than for either condition without protective clothing (relative to hat: t(15)=3.43, p = .004, d_Z_ = 0.857; relative to no hat: t(15)=2.40, p = .030, d_Z_ = 0.600). Error rates for the condition with the vest was lower than for any other condition (relative to hat: t(15)=3.83, p = .002, d_Z_ = 0.959, relative to no-hat: t(15)=2.89, p = .011, d_Z_ = 0.723, relative to hatband: t(15)=2.45, p = .027, d_Z_ = 0.612). This means that wearing a vest is more effective than wearing a hatband in terms of error reduction, but wearing a hatband is more effective than no protection.

Expressed in SDT measures, participants were well able to discriminate target-present from target-absent trials (mean d’: 4.39, range: 3.21–5.52). 11/16 observers applied a liberal criterion (rather wrongly reporting presence than absence), 5/16 a conservative criterion. The criterion c ranged from −0.40 to 0.29 and the mean of −0.10 was significantly smaller than 0 (t(15)=−2.35, p = .033), suggesting that observers were slightly more likely to incorrectly report the presence of a person than incorrectly reporting their absence.

We found a trend for orange vests to yield fewer errors (Fig 3B; mean: 0.16%, sd: 0.43%) than yellow vests (mean: 1.0%, sd: 1.9%). The fact that this trend did not reach significance (t(15)=1.90, p = .077, d_Z_ = 0.475) likely is due to a floor effect with 14 participants having no errors for the orange vest, and 10 participants for the yellow vest.

#### Reaction times.

As expected in visual search, median behavioral reaction times per observer for correct trials (RTs; Fig 3C) were about doubled in the target-absent trials (mean: 1.01s, sd: 0.43s) compared to the target-present trials (mean: 0.48s, sd: 0.09s). In target-present trials, RTs depended on the condition (F(3,45)=17.3, p < .001, ɳ^2^ = .535). There was only a trend to a difference between hat and no-hat (t(15)=2.02, p = .062, d_Z_ = 0.505), but any condition with protective clothing (hatband or vest) showed faster RTs than any condition without (all t(15)>3.06, all p < .008, all d_Z_ > 0.765). Interestingly, however, we did not observe a difference between hatband and vest (t(15)=0.92, p = .375, d_Z_ = 0.229). That is, if only behavioral reaction times were considered, responses were faster with protective clothing than without, but there was no difference between the levels of protective clothing used.

When comparing orange vests to yellow vests (Fig 3D), we found responses to oranges vests (mean: 0.45s, sd: 0.09s) to be faster than for yellow vests (mean: 0.47s, sd: 0.09s; t(15)=5.29, p < .001, d_Z_ = 1.32).

### Gaze

**Fig 4:**
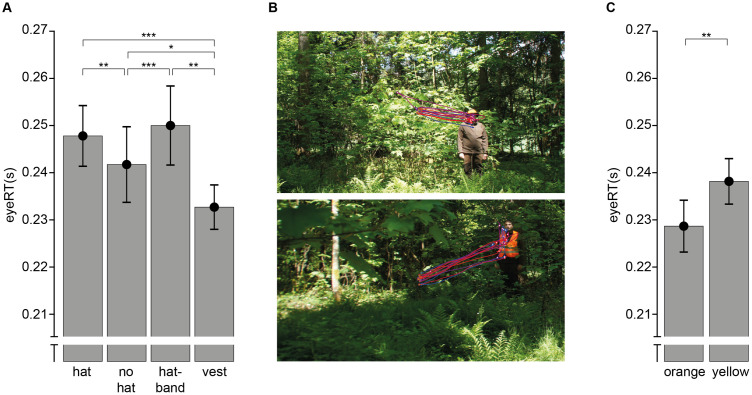
Gaze Data. **A)** Time to the first fixation on the person (eyeRT) by condition. **B)** Example for gaze data for two stimuli. Each colored line represents the gaze position data of a different individual, green and white filled circles fixations (green-first fixation, starting before stimulus onset). *Top:* hatband, *bottom:* vest. Note the frequent second saccades after fixating on the vest. **C)** eyeRT in the vest condition split by vest color. Bars denote mean and s.e.m. across individuals. Significance markers in all panels refer to pair-wise comparisons (*p < 0.05; **p < 0.01; ***p < 0.001). *The persons depicted in panel B agreed in writing to the unrestricted use of their image. All images have been made available by the authors under a CC-BY-4.0 license.*

In principle, the present/absent task could be solved without shifting gaze to the search target. Indeed, one participant fixated the person in only 5.5% of the target-present trials, while their error rate and RTs were well within in the range of the other observers. The other 15 observers fixated the target in at least 60% of target-present trials (mean: 89.9%, sd: 9.2%, range: 60.5%–99.7%) prior to their response. For these, we defined eye reaction times (eyeRT) as the time from stimulus onset till the participant fixated the target. As for behavioral RTs, we restricted analysis to correct trials and here also to trials in which the target was fixated. Of those trials, we computed the median for each participant and target-present condition. These eyeRTs depended on condition (F(3,42)=12.87, p < .001, ɳ^2^ = .479; Fig 4A). EyeRTs were indistinguishable between hat and hatband condition (t(14)=0.87, p = .400, d_Z_ = 0.224), which were both slower than the no-hat condition (both t(14) > 3.03, p < .009, d_Z_ = 0.784). Importantly, eyeRTs in the vest condition were not only faster than in conditions without protective clothing (vest vs. hat: t(14)=6.30, p < .001, d_Z_ = 1.63; vest vs. no-hat: t(14)=2.29, p = .038, d_Z_ = 0.592), but also substantially faster than in the hatband condition (t(14)=3.72, p = .002, d_Z_ = 0.959). That is, for eyeRTs vests show the expected benefit that was absent for behavioral RTs. A closer inspection of individual trials provides a potential explanation for this discrepancy: for the hatband condition, participants typically respond immediately after making the eye movement to the hatband, while in the vest condition, there is often another saccade to the face before the behavioral response is executed (Fig 4B). Indeed, analysis confirms this intuition: the average number of saccades between fixating the person and responding is 0.42 (sd: 0.24) in the hatband condition and 0.53 (sd: 0.30) in the vest condition (difference: t(14)=3.37, p = .005, d_Z_ = 0.870; inclusion of trials as in main gaze analysis). As for behavioral RTs, eyeRTs showed a benefit of orange over yellow vests (t(14)=3.30, p = .005, d_Z_ = 0.853; Fig 4C). In sum, the gaze-based analysis shows a clear benefit of the vest over all other conditions with respect to the time needed to first fixate the search target.

## Discussion

In a visual-search experiment, we compared different levels of protective clothing in forestial settings. We found that responses were faster and more accurate when the search targets wore protective (reflective) clothing. Reflective vests were more efficient than reflective hatbands in terms of search accuracy. For reaction times, this difference only manifested when eye movements were considered, but not in the behavioral data. Besides the central conclusion for the applied question – vests are better than hatbands, but hatbands are better than nothing – this also highlights a more abstract issue: there are cases where gaze tracking allows insights that are unavailable from behavioral data alone.

We found that models in orange vests were discovered faster than models in yellow vests. It is likely that this is a consequence of color contrast, as orange is more remote from green in any color space than yellow, and the local difference in color is one key component of a region’s salience [19,43]. It should be noted, however, that the effect of real-world luminance differences (contrasts), which are another contributor to visual salience, might be underestimated in any on-screen experiment compared to the real world, as the luminance range of a screen is necessarily limited compared to the several orders of magnitude of luminance variation in the wild, especially in the case of reflective surfaces.

It might be argued that reflective vests gain their advantage over hatbands by the larger area they cover, as size and apparent size play an important role in attention guidance – both in search arrays [30,32,33] and for objects in natural scenes [31,34]. While even for a random fixation distribution, larger objects would be fixated more than smaller objects, it should be noted that most models of salience in natural scenes are scale-invariant by design. For example, the saliency map [19] integrates over several layers of a Gauss-Laplace pyramid, such that a small, isolated object on a uniform background would yield the same model-based saliency as a large object. This, in turn, reflects the observation that natural scenes at large are scale invariant [44]. Even if the area of the vest contributes to its higher efficacy in attracting the first fixation compared to the hatband, this would still hold in practice and therefore be in line with the conclusion for the applied question. The fact that the vest’s larger area does *not* speed up the behavioral response compared to the hatband, in turn, highlights the non-trivial interplay between modelled physical salience, its actual impact on gaze and the resulting behavioral response.

Since we were primarily interested in the differences between the target-present conditions, we deliberately chose to present four times more target-present than target-absent trials. Low target prevalence yields an increased number of misses (present targets are not reported), which is mostly attributable to a shift in response bias (criterion) towards “absent” responses [45]. In turn, excessively high target prevalence can induce an increase in false alarms, which again is explained by a criterion shift without change in sensitivity [46]. Our target prevalence was still lower (80%) than the prevalence used in reference [46] (98%), and while *do* see a slightly liberal criterion on average, the 1.8% false alarm rate with a range from 0 to 5.0% is still far from excessive. The substantially longer search time for target-absent trials compared to target-present trials, is also typical for visual search experiments, but the about two-fold slowing observed here is not as extreme as in the 98% case of [46] and closer to their 50% prevalence cases. Hence, we consider it unlikely that the comparably high target prevalence had a critical impact on our results.

From a theoretical perspective, the key observation in the present study is the difference between data obtained from saccadic reaction times compared to data from behavioral reaction times. This is an example of a broader observation that eye-tracking data may reveal insights into visual search that could not be achieved by reaction times alone. In general, search strategies (and their failures), which would not be directly accessible from behavioral data, are often revealed by gaze data (see, e.g., references [35] and [47] for detailed accounts on benefits and challenges of using eye tracking in visual search). Consequently, eye tracking has provided fundamental insights into search strategies in many applied cases, such as medical image analysis [48,49], web search [50], driving instruction [51], military training [52] and education in general [53]. Eye-tracking data allows dissociating distinct phases of search (initiation, scanning, verification [36]), which again can be particularly relevant for applied cases. For example, in human-machine interaction, not executing a timely and appropriate response can result from difficulties in finding the relevant response element (failure of scanning) or in identifying it correctly (failure of verification); two types that require different mitigation strategies in human-machine interface design [54]. From an applied perspective, it would then also be relevant to see whether the present results, especially the difference between scanning and verification, extend to other types of gear and to structures designed to enhance visibility of inanimate objects, such as signposts marking obstacles on roads or in farmland, to inform and improve their design. While in our task, benefits of the vest are only revealed by gaze, the reverse can also hold. For example, alarm signals can be more salient in that they are fixated more quickly, but the same property that makes them salient can slow down the behavioral response rendering too much salience harmful [55]. In line with the beneficial use of eye tracking, the present study highlights a distinction between gaze and behavioral data. It thereby exemplifies how gaze and behavior can provide complementary information for assessing issues of real-life relevance.

### Limitations and outlook

While we took care to match the conditions as closely as possible, in particular with respect to the size and the distance of the search target, the precise location where a particular model is placed at the site can vary between somewhat between conditions. As such it is possible that – for a given model – their visibility is influenced by some incidental circumstances (such as the relative location of twigs, branches or other pieces of foliage). Since each model was photographed at a different location (but all conditions of the same model were roughly at the same site, such that the variability of the environment between models is far larger than between the different conditions of the same model), we consider it highly unlikely that there is any systematic relation between these incidental circumstances and the experimental condition; this is confirmed by visual inspection of the stimulus material. Hence, it is exceedingly unlikely that our results are influenced by incidental visibility effects, which, if anything, are an additional source of noise.

Obviously, there are many differences between a visual search task and the hunting scenario that motivated our study. For example, rather than giving a motor response in any case, the hunter needs to inhibit a motor action when a person suddenly appears in their target area or line of fire. Given that confusing game with a person is a relevant source of hunting accidents [4], identification might be a relevant aspect in addition to search and discrimination; considering the importance of biomotion markers in pedestrian recognition [9,10], using dynamic stimuli might therefore be an interesting future extension for the present line of work. Though not explicitly queried, most of our participants had likely little to no hunting experience and their age range might not be fully representative for the hunter population. Since age is a major factor for visual detection and recognition under poor visibility even when reflective materials are worn [11], a broader sample may improve the representativeness of our study. Finally, no rapid responses are typically required during hunting and in the real world, there are also other modalities available to indicate approaching persons, in particular auditory cues. Nonetheless, for the question of easy visibility visual search is likely a good proxy. Moreover, visibility in forestial environments might also be useful beyond the hunting context, for example, to prevent logging accidents or for aiding search and rescue when getting lost or incapacitated in the forest. As such, we consider the present study a general basic research foundation, whose results and importantly its database may serve as a starting point for research on various more specific applied scenarios.

Our forest scenes differ from scenes used in typical real-world visual search tasks in that they contain few surfaces, few objects and in some even the ground is not quickly discernable. This limits contextual guidance that is otherwise observed in natural scene search [18,38]. By placing the search target at a random location relative to the screen (except too close to the center) and by using each image also in its mirror reversed version, we further limit any use of image-independent search strategies and effects of general search biases. Comparing search in our foliage scenes to more structured, but equally cluttered scenes (e.g., an urban street scene or people on forest roads) might therefore provide additional insight into the extraction of scene structure and its effect on search and target visibility. From an applied perspective, such research may link the present question of visibility in the forest to related issues such as visibility of workers or vulnerable road users in traffic [12,13].
